# Cryoablation Versus Radiofrequency Ablation in the Management of Pediatric Supraventricular Tachyarrhythmia: A Systematic Review and Meta-Analysis

**DOI:** 10.7759/cureus.77812

**Published:** 2025-01-22

**Authors:** Dost Jabarkhyl, Muhammad Khursheed Ullah Khan Marwat, Naveed Haider, Aala Farah, Manaf Yusuf, Nasir Ali, Waqar Aziz

**Affiliations:** 1 General Internal Medicine, Luton and Dunstable University Hospital, Luton, GBR; 2 Infectious Diseases, Hull University Teaching Hospitals NHS Trust, Hull, GBR; 3 Pediatrics, Sheffield Children’s Hospital, Sheffield Children’s NHS Foundation Trust, Sheffield, GBR; 4 Pediatrics, Leeds Children’s Hospital, Leeds Teaching Hospitals NHS Trust, Leeds, GBR; 5 Cardiac Imaging, St George's University Hospitals NHS Foundation Trust, London, GBR

**Keywords:** ablation, catheter ablation, cryoablation, pediatric arrhythmia, radiofrequency, supraventricular tachycardia

## Abstract

Supraventricular tachycardia (SVT) is a common arrhythmia in pediatric patients, often requiring catheter ablation for effective treatment. Two primary techniques, radiofrequency ablation (RFA) and cryoablation (CA), are widely used; however, their comparative safety and efficacy remain subjects of debate, with no clear consensus on the preferred approach. This systematic review and meta-analysis aimed to evaluate and compare the efficacy and safety of RFA and CA in pediatric patients with SVT, focusing on the primary outcomes of acute success and recurrence rates. The study was conducted following the Preferred Reporting Items for Systematic Reviews and Meta-Analyses (PRISMA) guidelines. Eligible studies included comparative analyses such as randomized controlled trials, non-randomized trials, and observational studies that specifically evaluated RFA and CA in pediatric populations with SVT. Exclusion criteria included studies involving adult populations, those without comparative groups, case reports, case series, and conference abstracts. Data extracted from the included studies encompassed acute success rates, recurrence rates, and complication rates, providing a comprehensive overview of the performance and safety profiles of RFA and CA in this patient group. Acute success rates were high for both techniques (RFA: 96.3%, CA: 94.9%; p = 0.137). However, RFA demonstrated a significantly lower recurrence rate (7.9% vs. 14.4%; odds ratio (OR): 0.408, 95% CI: 0.242-0.689, p < 0.001). CA was associated with longer procedure durations (mean difference: 9.684 minutes, p = 0.437) and significantly reduced fluoroscopy times (mean difference: 6.566 minutes, p = 0.032). Complication rates were comparable, with a non-significant trend favoring RFA (OR: 0.363, p = 0.112). Overall, both RFA and CA were found to be effective and safe for pediatric SVT. RFA offers durable results with lower recurrence rates, while CA minimizes fluoroscopy time, thereby reducing radiation exposure. Treatment selection should be individualized, considering factors such as the type and location of the arrhythmia as well as specific procedural risks.

## Introduction and background

Arrhythmias in the pediatric population can significantly impact patient quality of life, potentially leading to complications such as heart failure, syncope, and an increased risk of sudden cardiac death. While pharmacotherapy can be an effective management strategy, catheter ablation has increasingly become the preferred treatment for many pediatric patients due to its potential for curative outcomes and lower recurrence rates [1]. The two primary ablation techniques - radiofrequency ablation (RFA) and cryotherapy ablation (CA) - each have their unique advantages and associated risks, leading to an ongoing debate about their optimal use in children with arrhythmias.

RFA is widely used for treating various pediatric arrhythmias, particularly supraventricular tachycardias (SVTs) like atrioventricular nodal reentrant tachycardia (AVNRT), atrioventricular (AV) reentrant tachycardia (AVRT) associated with Wolff-Parkinson-White (WPW) syndrome, atrial ectopic tachycardia, and certain ventricular arrhythmias such as idiopathic ventricular tachycardia [2]. The procedure involves delivering high-frequency electrical energy to targeted cardiac tissue, generating localized heat-induced lesions that disrupt abnormal electrical pathways. Success rates of RFA in treating SVTs in children are high, often exceeding 90% [3]. However, the technique carries certain risks, especially given the smaller and developing cardiac structures in pediatric patients and delivering ablations closer to sensitive areas such as the AV node.

RFA complications include the risk of damage to the cardiac conduction system, potentially leading to heart block, which may necessitate permanent pacemaker implantation [4]. Additionally, RFA can cause scar tissue formation at the ablation site, occasionally resulting in proarrhythmia, where new arrhythmic foci are generated over time. Other potential complications, though rare, include coronary artery damage and pericardial effusion, particularly when ablating near vital structures like the coronary sinus or within the left atrium [5]. These risks highlight the importance of careful procedural planning, particularly for arrhythmias close to the AV node, as in the case of AVNRT.

CA, on the other hand, has gained popularity due to its favorable safety profile. CA is commonly used for AVNRT and accessory pathway-mediated tachycardias (e.g., AVRT), particularly when the arrhythmogenic substrate is close to sensitive regions such as the AV node [6,7]. The procedure involves freezing techniques to create lesions in cardiac tissue, with the ability to "cryomap" or create reversible lesions to test the effects before permanent ablation. This unique feature can reduce the risk of complications like permanent AV block [8]. While CA generally has a lower risk profile (compared to RFA), it is not entirely free of concerns. One of the main challenges is its potentially lower efficacy and higher recurrence rates, as cryo-induced lesions may be less durable than those created by RFA [7]. Although CA is associated with a reduced risk of permanent AV block due to its self-limiting nature, transient AV block can still occur during the procedure [9]. Other complications include vascular injury, phrenic nerve palsy (particularly when ablating near the superior vena cava), and rare occurrences of coronary artery spasm [10].

Choosing between RFA and CA often depends on the type of arrhythmia, (its) location, and the (individual) patient's anatomy [11]. RFA is generally preferred for its high efficacy and long-term success, especially for arrhythmias located away from critical conduction pathways. In contrast, CA is favored in scenarios where arrhythmias, like AVNRT, involve delicate regions near the AV node due to its lower risk of permanent conduction block [12]. Despite the growing use of both RFA and CA in pediatric patients, there is still no clear consensus on which approach offers the best balance of efficacy and safety. Existing studies often have small sample sizes, varying patient demographics, and different definitions of success and complications, making it challenging to draw firm conclusions.

Hence, there is a compelling need for a comprehensive meta-analysis to compare the efficacy, safety, and long-term outcomes of RFA and CA in the pediatric population. Through systematic data pooling from these studies, this analysis aims to elucidate the relative benefits and risks associated with each ablation technique, providing invaluable insights for clinicians tasked with managing pediatric arrhythmias.

## Review

Methodology

Methods

A systematic review and meta-analysis were conducted according to the Preferred Reporting Items for Systematic Reviews and Meta-Analyses (PRISMA) guidelines [13].

Eligibility Criteria

All comparative studies including randomized and non-randomized controlled trials and observational studies comparing RFA with CA for the treatment of SVT in pediatric age groups were included. Only articles reported in English were considered to meet the eligibility criteria.

Exclusion Criteria

Studies without a comparative group, those involving adult patients, case reports, case series, and abstracts were excluded from the selection process.

Primary and Secondary Outcomes

The primary outcomes of this meta-analysis were acute success rates and rates of recurrence. The secondary outcomes for this review were procedure time, fluoroscopy time, and rate of complications.

Literature Search Strategy

Three authors (NA, NH, and MY) independently searched the electronic databases MEDLINE, PubMed, ScienceDirect, and Cochrane Central Register of Controlled Trials (CENTRAL). The last search was run on September 21, 2024. In addition, the World Health Organization International Clinical Trials Registry (http://apps.who.int/trialsearch/), ClinicalTrials.gov (http://clinical-trials.gov/), and the ISRCTN registry (http://www.isrctn.com/) were also searched to identify details of any unpublished studies. The search terms included “radiofrequency”, “trans-catheter ablation”, “cryoablation”, “paediatric supraventricular”, “AVNRT”, “AVRT”, “atrioventricular nodal reentrant” and “tachyarrhythmia” The search terms were collated with adjuncts of “and” as well as “or”. The authors also searched the reference lists of the relevant articles to optimize screening and selection.

Selection of Studies

Two authors (MM and DJ) independently assessed the titles and abstracts of articles retrieved from the literature. Those meeting the eligibility criteria underwent a thorough full-text review. Studies that satisfied the criteria were subsequently selected for inclusion. Any discrepancy in study selection was resolved by NA who acted as an adjudicator.

Data Extraction and Management

Microsoft Excel was systematically used to develop an electronic data extraction spreadsheet, which was then adapted to align with Cochrane’s data collection form for intervention reviews. To ensure its effectiveness, the spreadsheet underwent a pilot test, where it was employed to extract data from a selection of random articles. Adjustments were made as needed to enhance the tool’s functionality and accuracy. The data collected included the name of the study, year of publication, total number of patients in each group (radiotherapy ablation and cryotherapy ablation), type of arrhythmia, complication rate, fluoroscopy time, recurrence rate, acute success, and procedure time. All data presented as median (IQR) were converted to mean (SD) using an online calculator [14]. Data extraction and recording were performed independently by six authors - DJ, NH, MY, NA, and AY - whilst another author, MM, was consulted on any uncertainties that arose.

Methodological Quality and Risk of Bias Assessment

The methodological quality as well as the risk of bias for randomized control trials that met the eligibility criteria was assessed with Cochrane’s Collaboration tool. Domains included in this evaluation were selection, performance, detection, attrition, and reporting bias as well as other sources. Studies were subsequently divided into low, unclear, or high bias. The Newcastle-Ottawa Scale was used to assess the methodological quality of all observational studies with domains assessing selection, comparability, and exposure [15]. The scale uses a scoring system with a maximum total score of nine stars for each study.

Data Synthesis

Open Meta-analyst and Microsoft Excel were used to conduct the data synthesis. All analyses were based on a random effects model and results were reported in forest plots with 95% confidence intervals (CI). For continuous outcome data, the mean difference was used to assess both groups and dichotomous outcomes were analyzed with odds ratios (ORs).

Assessment of Heterogeneity

Heterogeneity among the studies was assessed using the Cochran Q test (χ^2^) as well as calculating the I^2^ score, which was interpreted using the following scale: 0%-25% (low heterogeneity); 25%-75% (moderate heterogeneity); and 75%-100% (considerable heterogeneity).

Results

Literature Search Results

**Figure 1:**
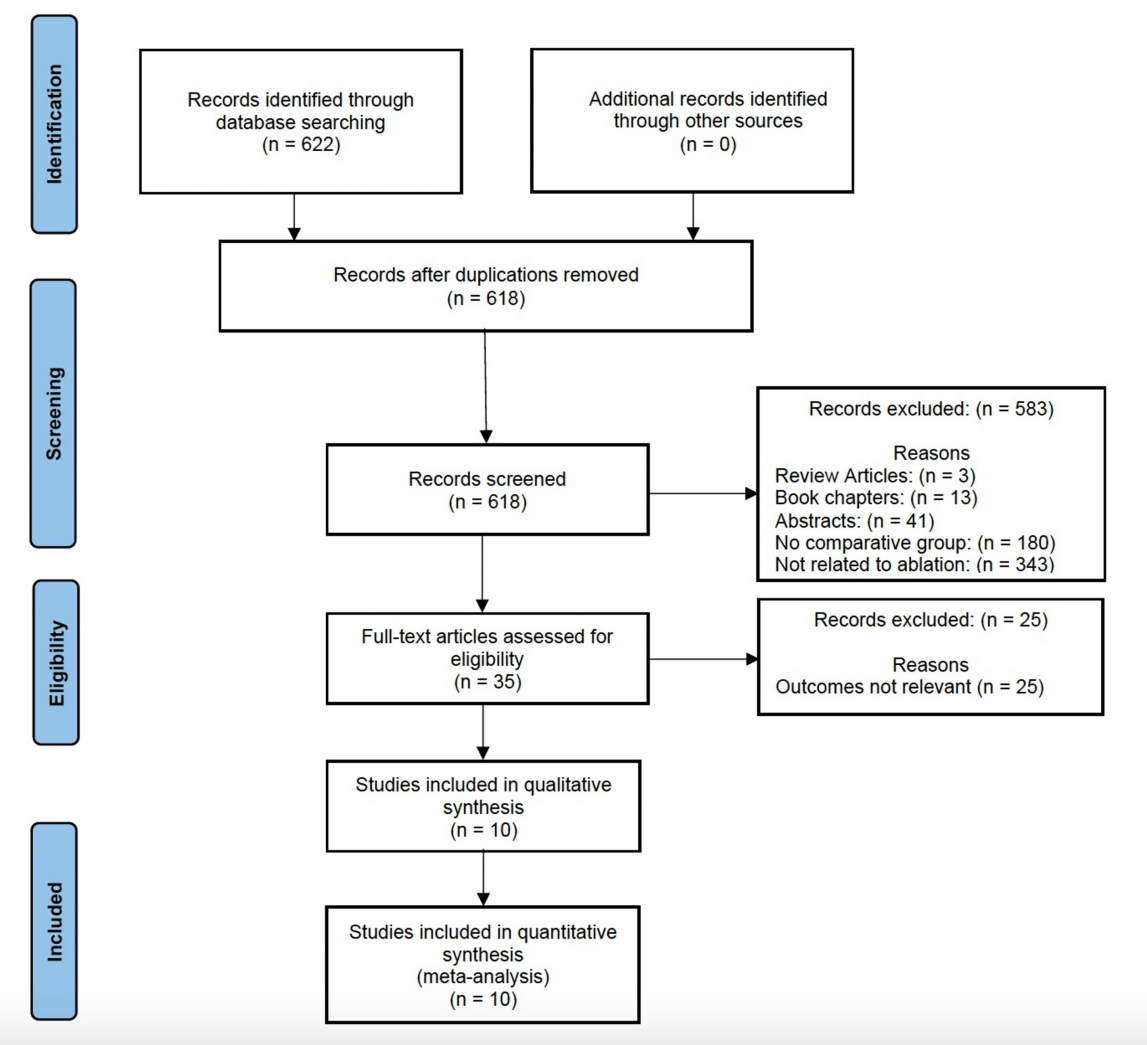
PRISMA flow diagram detailing the search and selection process PRISMA: Preferred Reporting Items for Systematic Reviews and Meta-Analyses

A total of 618 articles were retrieved through a literature search and screened by three authors (NH, NA, MY). Of these, 10 studies met the eligibility criteria for this systematic review and quantitative analysis [5,7,16-23]. A random effects model was used to analyze pooled data from these 10 studies (Figure 1).

Ten studies were selected based on eligibility criteria, and their baseline characteristics are summarized in Table 1.

**Table 1:** Baseline characteristics of included studies CA: cryoablation; RFA: radiofrequency ablation; N/R: not reported; AVNRT: atrioventricular nodal reentrant tachycardia; AVRT: atrioventricular reentrant tachycardia; M: male; F: female; n: number of cases ± indicates the standard deviation

Study (year)	Study design	Ablation type	Sex M:F (n)	Age: (years)	Sample size (n)	Type of SVT (n)	Success rate (%)	Recurrence rate	Fluoroscopy time (minutes)	Procedure duration (minutes)	Complications rate (%)
Papagiannis et al. (2010) [23]	Retrospective observational study	RFA	12:8	13.25 ± 2.59	20	AVNRT = 20	100%	2/20	10.9 ± 6.46	147.75 ± 37.15	5%
						AVRT = 0					
						Others = 0					
		CA	11:9	12.17 ± 3.07	20	AVNRT = 20	90%	5/18	6.41 ± 6.92	184.4 ± 75.59	5%
						AVRT = 0					
						Others = 0					
Krause et al. (2020) [19]	Prospective observational study	RFA	N/R	N/R	643	AVNRT = N/R	96%	43/573	N/R	N/R	N/R
						AVRT = N/R					
						Others = N/R					
		CA	N/R	N/R	40	AVNRT = 15	90%	4/33	N/R	N/R	N/R
						AVRT = 21					
						Others = 4					
Oster et al. (2016) [21]	Retrospective single center	RFA	N/R	13.55 ± 3.64	42	AVNRT = 42	83%	3/42	N/R	246 ± 41.84	N/R
						AVRT = 0					
						Others = 0					
		CA	N/R	13.01 ± 3.37	48	AVNRT = 48	100%	10/48	N/R	210 ± 40.82	N/R
						AVRT = 0					
						Others = 0					
Papez et al. (2006) [22]	Retrospective observational study	RFA	39:34	N/R	73	AVNRT = 60	96%	7/70	39 ± 25	248 ± 63	0%
						AVRT = 13					
						Others = 0					
		CA	37:44	13.4 ± 3.9	67	AVNRT = 52	94%	8/63	19.6 ± 24.5	237.2 ± 77	24%
						AVRT = 10					
						Others = 5					
Koca et al. (2022) [18]	Retrospective observational study	RFA	15:14	14 ± 0.75	29	AVNRT = 29	100%	2/29	N/R	86.67 ± 45.8	0%
						AVRT = 0					
						Others = 0					
		CA	18:48	14 ± 0.95	60	AVNRT = 60	100%	4/60	N/R	156.1 ± 37.7	33%
						AVRT = 0					
						Others = 0					
Chen et al. (2012) [5]	Retrospective observational study	RFA	4:16	13 ± 3	20	AVNRT = 20	100%	1/20	38 ± 18	187 ± 59	15%
						AVRT = 0					
						Others = 0					
		CA	9:9	16 ± 3	18	AVNRT = 18	100%	0/18	31 ± 13	172 ± 48	0%
						AVRT = 0					
						Others = 0					
Collins et al. (2006) [17]	Retrospective observational study	RFA	31:29	14 ± 4	60	AVNRT = 60	100%	1/53	21± 15	112 ± 32	10%
						AVRT = 0					
						Others = 0					
		CA	25:32	14 ± 4	57	AVNRT = 57	95%	4/49	20± 13	148 ± 46	11%
						AVRT = 0					
						Others = 0					
Noten et al. (2021) [20]	Retrospective observational study	RFA	90:94	14.75 ± 1.13	184	AVNRT = 51	97.8%	17/184	N/R	85.13 ± 11.31	1.1%
						AVRT = 133					
						Others = 0					
		CA	19:20	14.25 ± 1.13	39	AVNRT = 28	84.61%	16/39	N/R	118.89 ± 13.67	2.6%
						AVRT = 11					
						Others = 0					
Avari et al. (2007) [16]	Retrospective observational study	RFA	14:28	12.2 ± 4.75	42	AVNRT = 42	95.4%	1/41	21 ±38.5	214.75 ± 93.75	0%
						AVRT = 0					
						Others = 0					
		CA	11:27	13.3 ± 3.4	38	AVNRT = 38	97.4%	1/36	19 ± 10.75	170.75 ± 34.25	24.3%
						AVRT = 0					
						Others = 0					
Buddhe et al. (2012) [7]	Retrospective observational study	RFA	N/R	13.4 ± 3.7	87	AVNRT = N/R	98%	12/85	N/R	N/R	0%
						AVRT = N/R					
						Others = N/R					
		CA	N/R	N/R	9	AVNRT = N/R	100%	2/9	N/R	N/R	0%
						AVRT = N/R					
						Others = N/R					

Risk of Bias Assessments

The Newcastle-Ottawa Scale [15] was used to assess the quality of the studies, all of which achieved good scores as shown in Table 2.

**Table 2:** Newcastle-Ottawa Scale quality assessment of included studies The evaluation consists of three categories: selection (maximum of four stars (****)), comparability (maximum of three stars (***)), and outcome (maximum of two stars (**)). The table displays the actual scores achieved by the studies in each category, which may include no stars if the study did not meet the relevant criteria. The single star (*) indicates that the study met one specific criterion within the respective category as assessed by the Newcastle-Ottawa Scale. For selection, stars were awarded for the following criteria: representativeness of the average pediatric community, the non-exposed cohort drawn from the same community, data extracted from secure records, and absence of the outcome of interest at the start of the study. For comparability, stars were awarded if the study controlled for age between groups and for congenital heart disease (CHD). Avari et al. 2008, Krause et al. 2020, and Koca et al. 2022 controlled for both age and CHD [16,18,19], while Chen et al. 2012 reported a high prevalence of CHD in the cryotherapy group [5]. In the outcome category, stars were awarded if results were reported from secure records, if all participants had a one-year follow-up, and if at least 85% of participants were followed up. Rating scale: 7-9 stars = low risk of bias; 4-6 stars = moderate risk of bias; 0-3 stars = high risk of bias

Study	Selection	Comparability	Outcome
Collins et al., 2006 [17]	***		**
Papez et al., 2006 [22]	***	*	*
Avari et al., 2008 [16]	***	**	*
Buddhe et al., 2012 [7]	***	*	*
Chen et al., 2012 [5]	***	*	**
Papagiannis et al., 2010 [23]	***	*	**
Oster et al., 2016 [21]	***	*	*
Krause et al., 2020 [19]	***	**	**
Noten et al., 2021 [20]	***	*	**
Koca et al., 2022 [18]	***	**	**

Primary outcomes

Success Rate

**Figure 2:**
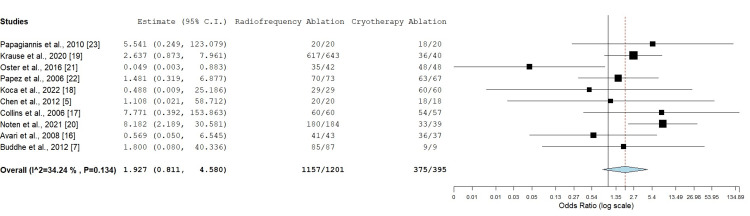
Success rates comparison between radiofrequency ablation and cryotherapy ablation Odds ratio: 1.927 (95% CI: 0.811-4.580), p = 0.137, I² = 34.24% CI: confidence interval

All the included studies measured the acute success rate of the procedures (n = 1596), a total of 1201 patients received RFA while 395 patients received CA. About 1157 out of 1201 patients who received RFA achieved acute success (96.3%) while 375 out of 395 patients who underwent CA achieved acute success (94.9%). Statistical analysis showed that there was no statistically significant difference in success rates between CA and RFA (OR: 1.927 (0.811 to 4.580), p = 0.137)). Moderate heterogeneity was observed (I² = 34.24%, p = 0.134), indicating some variability between studies (Figure 2).

Recurrence

**Figure 3:**
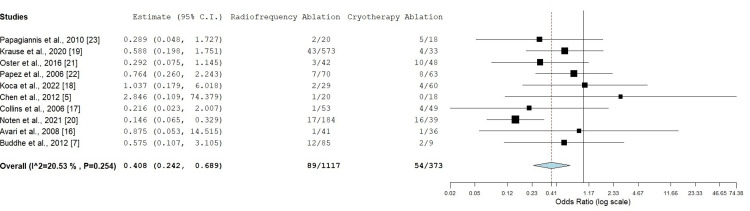
Recurrence rate between radiofrequency ablation and cryotherapy ablation in pediatric patients Odds ratio: 0.408 (95% CI: 0.242-0.689), p < 0.001, heterogeneity: I² = 20.53%, p = 0.254 CI: confidence interval

Of the 1,532 patients who achieved acute procedural success, 1,490 were followed up for the recurrence of SVT, while 42 patients were lost to follow-up. Follow-up periods of patients in these studies varied widely with mean (SD) follow-up found to be 23.52 ± 31.54 months. Among the 1,117 patients who underwent successful RFA and were followed up, 89 experienced a recurrence, resulting in a recurrence rate of 7.9%. In comparison, 54 out of 373 patients who underwent successful CA and were followed up experienced recurrence, corresponding to a recurrence rate of 14.4%. The analysis showed that RFA was associated with a lower recurrence rate in comparison to CA in six studies, with an OR of 0.408 (95% CI: 0.242-0.689) (Figure 3) [7,15,17,19,20,21]. This result approached statistical significance (p < 0.001) and exhibited low heterogeneity (I² = 20.53%), suggesting consistency across the studies. This finding is consistent with prior research, suggesting that RFA may provide a more enduring form of arrhythmia management.

Secondary outcomes

Procedure Duration

**Figure 4:**
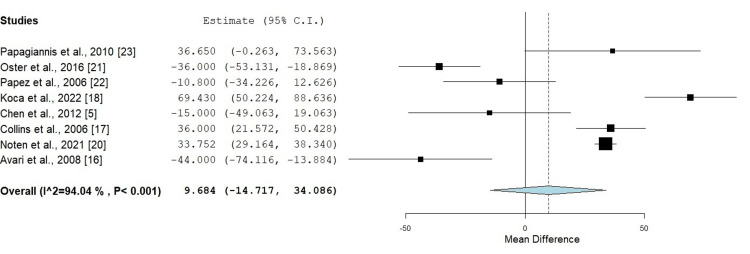
Comparison of procedure duration (minutes) between radiofrequency ablation and cryotherapy ablation in pediatric patients Mean difference: 9.684 (p = 0.437), heterogeneity: I² = 94.04%, p < 0.001

Eight out of 10 studies measured procedure duration (n = 817). The results indicated that CA had a longer mean procedure duration than RFA, with a mean difference (MD) of 9.684 minutes. This finding, however, was not statistically significant (p = 0.437) and showed significant heterogeneity among the studies (Figure 4).

Fluoroscopy Time

**Figure 5:**
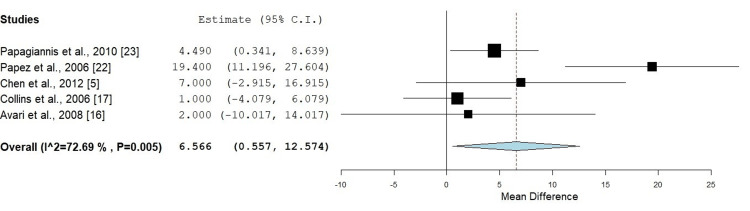
Comparison of fluoroscopy time between radiofrequency ablation and cryotherapy ablation in pediatric patients Mean difference: 6.566 minutes (95% CI: 0.557-12.574), p = 0.032, Heterogeneity: I² = 72.69%, p = 0.005 CI: confidence interval

Six out of 10 included studies included fluoroscopy time; however, Krause et al. mentioned a combined fluoroscopy time for RFA and CA; therefore, only five studies were included in the analysis (n = 387) [5,16,17,22,23]. The pooled mean difference was 6.566 minutes (95% CI: 0.557-12.574), favoring cryotherapy, indicating that cryotherapy procedures may require less fluoroscopy time than RFA. The p-value for this comparison was 0.032, signifying statistical significance and suggesting a potentially relevant clinical benefit in reducing radiation exposure during cryotherapy ablation procedures in pediatric patients (Figure 5). The heterogeneity of the included studies was moderate, with an I² value of 72.69%, p = 0.005, indicating some variability between the study results (Figure 5).

Complications

**Figure 6:**
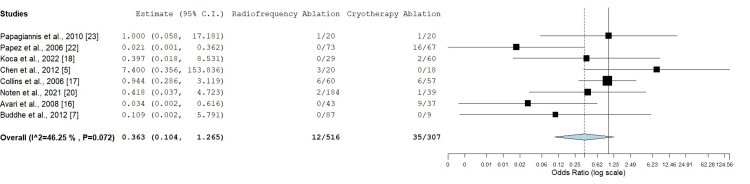
Comparison of complication rates between radiofrequency ablation and cryotherapy ablation in pediatric patients Odds ratio: 0.363 (95% CI: 0.104-1.265), p = 0.112, heterogeneity: I² = 46.25%, p = 0.072 CI: confidence interval

Nine out of 10 studies included complication rates; however, Krause et al. described a combined complication rate for both RFA and CA; therefore, eight studies were included in the analysis of this outcome (n = 823) [5,7,16-20,22,23]. Statistical analysis estimates an OR of 0.363 (95% CI: 0.104-1.265), suggesting that RFA may have a lower complication rate than cryotherapy. However, the CI crosses 1.0, and the p-value is 0.112, indicating that the result is not statistically significant. The heterogeneity of the included studies was moderate, with an I² value of 46.25% reflecting moderate variability in complication rates across studies (Figure 6).

Discussion

RFA and cryoablation (CA) have become pivotal in managing arrhythmias in pediatric patients, including atrioventricular nodal re-entrant tachycardia (AVNRT), atrioventricular re-entrant tachycardia (AVRT), atrial flutter, and focal atrial tachycardia. RFA became the standard for treating arrhythmias due to its high success rates and durability, demonstrating significant improvements in patient outcomes [24]. The procedure involves delivering targeted thermal energy to the heart tissue, creating lesions that disrupt the electrical pathway responsible for the arrhythmia [24]. Despite its efficacy, the risk of inadvertent damage to nearby structures, especially the AV node, presents a concern, particularly in pediatric patients with smaller cardiac anatomy. This risk includes iatrogenic complete heart block, which may necessitate lifelong pacemaker implantation [25].

CA emerged as a safer alternative, particularly for patients with arrhythmias located near critical structures. By using cryothermal energy to create reversible, transient lesions, CA allows for safer mapping of areas near sensitive conduction tissue. This "test freeze" capability significantly reduces the risk of AV block, making CA particularly appealing for pediatric cases involving the AV node [22]. Despite these safety advantages, studies have suggested that CA may result in higher recurrence rates due to the less permanent nature of the lesions it creates [26].

This meta-analysis sought to compare the outcomes of RFA and CA across a variety of pediatric arrhythmias. The key outcomes included acute success rates, recurrence rates, procedure times, fluoroscopy times, and complications. The results of this analysis provide insights into the efficacy and safety profiles of both ablation modalities. The acute success rates analyzed in this study showed a trend favoring RFA (96.3%) over CA (94.9%), though the difference was not statistically significant (p = 0.137). This trend aligns with the findings of Papagiannis et al. (2010), who reported slightly higher success rates for RFA in treating AVNRT in pediatric patients [23]. The safety profile of CA, which allows the creation of reversible lesions, is especially beneficial for arrhythmias situated near sensitive structures like the AV node since these test lesions are reversible in case they cause AV block during cryo-mapping. Moreover, cryo-adhesion allows the catheter tip to remain in position allowing for more accurate lesion formation [6]. This advantage, however, comes with a trade-off, as evidenced by the slightly lower success rates observed in some studies [19,20,22,23].

A significant finding of this meta-analysis is the difference in recurrence rates between the two modalities. RFA demonstrated a lower recurrence rate (OR of 0.370, p < 0.001), suggesting more durable arrhythmia control compared to CA. This aligns with the findings of previous studies that reported higher recurrence rates associated with CA, likely due to the less permanent nature of cryothermal lesions [5,19,21,23]. The long-term efficacy of RFA in reducing recurrence has also been documented in other studies, extending its benefits to arrhythmias like AVRT and focal atrial tachycardia. Conversely, while the reversible lesions created by cryotherapy enhance safety, they may contribute to a higher likelihood of arrhythmia recurrence, potentially requiring subsequent interventions [23,24]. Similarly, meta-analyses have indicated that the recurrence rates of RFA for septal accessory pathways are relatively low compared to CA in the adult population, as shown in the study by Bravo et al. (2018) [27].

When analyzing secondary outcomes, such as procedure duration and fluoroscopy time, this meta-analysis revealed that although the procedure duration associated with CA was longer (mean difference: 9.684 minutes), this difference was not statistically significant (p = 0.437). However, CA demonstrated a significantly shorter fluoroscopy time (mean difference: 6.566 minutes, p = 0.032). This finding holds particular significance in pediatric patients, as minimizing radiation exposure is crucial in this population. Santangeli et al. (2021) similarly concluded that CA could reduce fluoroscopy exposure, thereby mitigating long-term radiation-associated risks [26].

Complication rates are a critical consideration in pediatric ablation procedures. The current analysis indicated a trend toward a lower complication rate with RFA (OR: 0.363), although this difference was not statistically significant (p = 0.112). CA, with its ability to produce reversible lesions, has been associated with a lower risk of major complications such as AV block, a significant concern in pediatric patients [23]. Low rates of AV block are reported in the adult population RFA in comparison to CA for septal accessory pathway [27].

In regard to the limitations of the study, all included studies were observational, and all but one were retrospective. However, they achieved good scores on the methodological quality assessment using the Newcastle-Ottawa scale. The study included a robust sample size of 1,596 patients, providing consistency in the primary and secondary outcome measures. The results of the quantitative review are not statistically significant apart from the recurrence rate and fluoroscopy time with recurrence rates lower in the RFA compared to CA while shorter fluoroscopy times in CA compared to RFA. There were certain limitations of the study which included considerable heterogeneity among studies for procedure duration and moderate heterogeneity among studies comparing fluoroscopy time; however, this was circumvented by using the random effects model in the analysis. Additionally, the inclusion of only English language articles introduces language bias, potentially excluding relevant studies published in other languages, which may have influenced the findings. The authors recommend the conduction of high-quality randomized control trials to provide more evidence on the effectiveness of RFA versus CA. Due to the vast diversity and anatomical complexity of different arrhythmia types, clinical trials with more categorization of patients based on different subtypes of arrhythmias would provide more effective evidence for the preference of one technique over the other.

## Conclusions

This meta-analysis indicates that both RFA and cryotherapy are effective and safe options for managing arrhythmias in pediatric patients. The main difference between the procedures, however, was statistically significant lower arrhythmia recurrence in the RFA group. The decision between these modalities should be individualized, taking into account the specific arrhythmia type, location, patient age, and potential risks associated with each procedure.
